# Supplementary material for: Basigin links altered skeletal stem cell lineage dynamics with glucocorticoid-induced bone loss and impaired angiogenesis
Source: Nat Commun. 2025 Aug 15;16:7606. doi: 10.1038/s41467-025-62881-w (PMC12356905; doi:10.1038/s41467-025-62881-w)
Supplement: Supplementary file 1 — Supplementary Information [file 41467_2025_62881_MOESM1_ESM.pdf]

Supplementary Figure 1

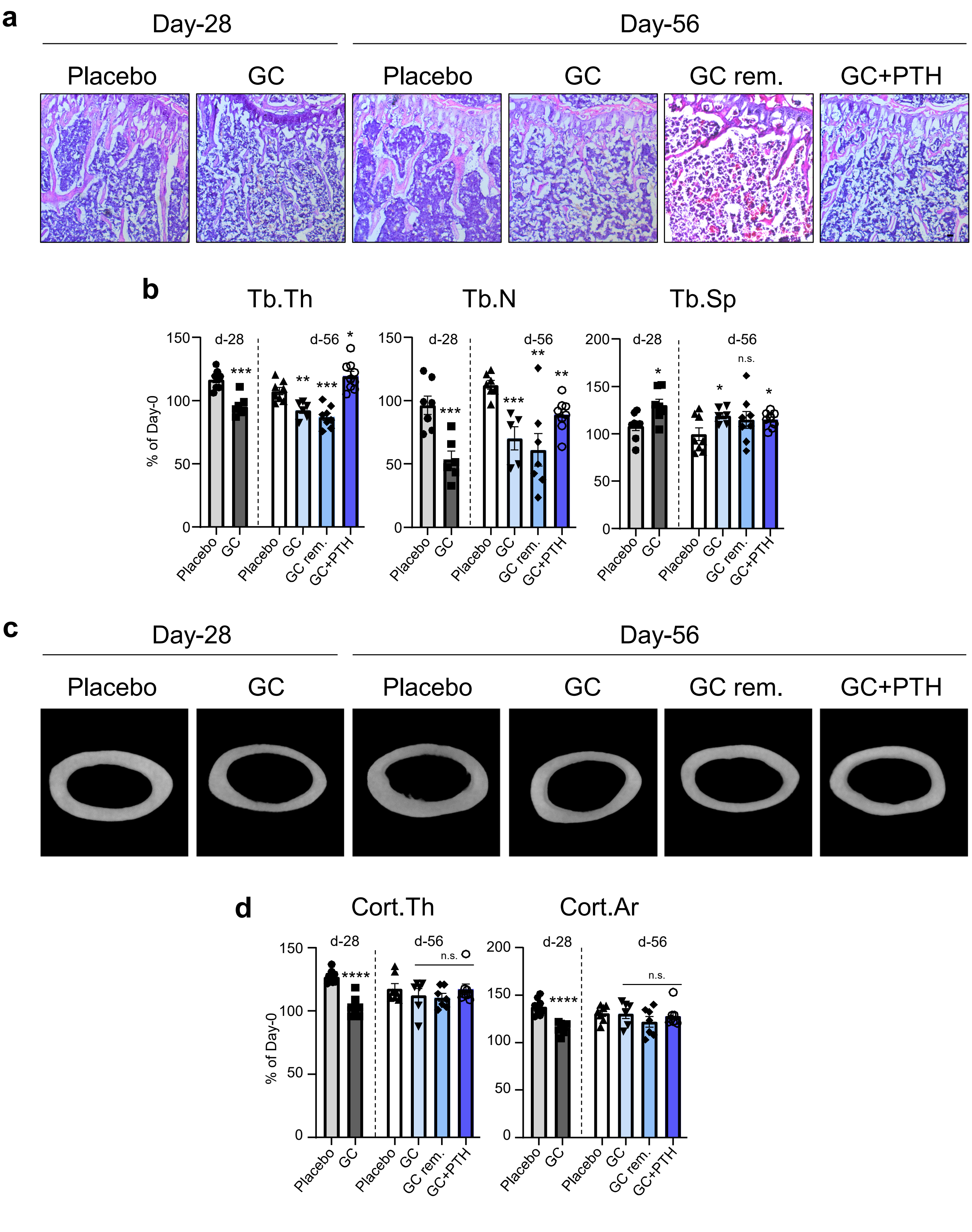

Supplementary Figure 1. Micro-CT bone parameters of experimental groups. (a) Representative H&E staining images of metaphyseal regions of distal femurs of different experimental groups. **(b)** Quantification of trabecular bone thickness (Tb.Th), number (Tb.N) and spacing (Tb.Sp) shown as percentage change compared to day 0. Analysis of femur bones from n=8 biologically independent mice for Placebo and GC removal groups, n=7 for GC d-28, n=6 for GC d-56 and n=9 for GC+hPTH. **(c)** Representative microCT images of cortical bone at day-28 and day-56. **(d)** Quantification of femoral cortical thickness (Cort.Th) and cortical area (Cort.Ar.). Analysis of femur bones from n=8 biologically independent mice for Placebo d-28 and GC+hPTH groups, n=7 for GC d-28, Placebo d-56 and GC removal groups, n=6 for GC d-56. Statistical testing between Placebo and other group by two-sided unpaired student t-test. *$p<0.05$, **$p<0.01$, ***$p<0.001$, ****$p<0.0001$. Scale bars, 50 μm.

Supplementary Figure 2

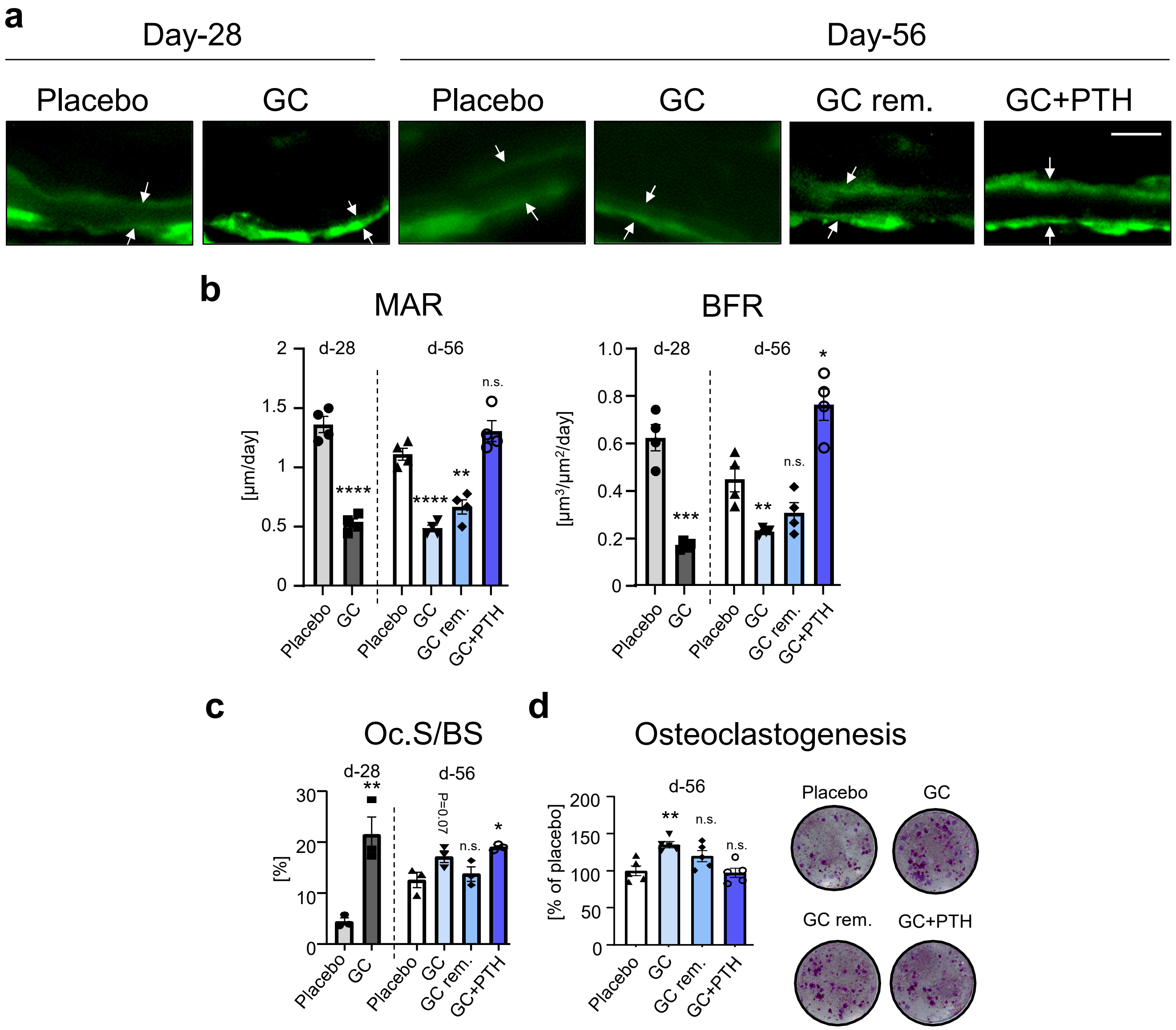

Supplementary Figure 2. Bone parameters of experimental groups. (a) Representative Calcein double labeling for each experimental group. **(b)** Quantification of mineral apposition rate (MAR) and bone formation rate (BFR) based on Calcein double labeling. n=4 biologically independent mice per group. **(c)** Quantification of osteoclast surface per bone surface (OC.s/BS) by TRAP labeling. n=3 biologically independent mice per group. **(d)** Quantification of bone marrow derived osteoclast formation from bones harvested at day-56 (left) and representative images thereof (right). Bone marrow-derived cells from n=5 biologically independent mice per group. All data shown as mean ± SEM. Statistical testing between Placebo and other group by two-sided unpaired student t-test. *p<0.05, **p<0.01, ***p<0.001, ****p<0.0001. Scale bar, 50µm.

Supplementary Figure 3

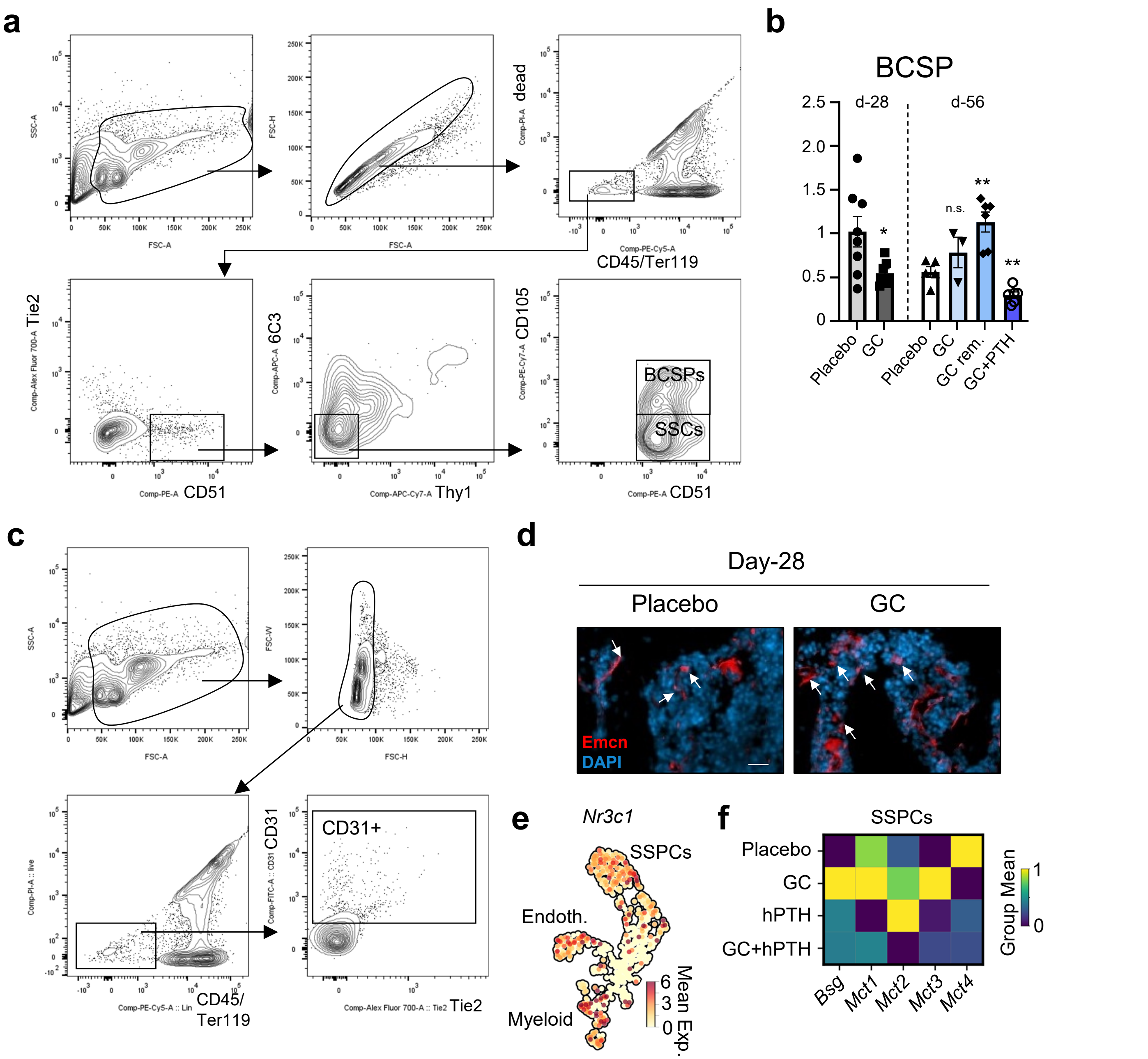

Supplementary Figure 3. Flow cytometric gating strategies. (a) Representative gating strategy for skeletal stem cell (SSC) and bone-cartilage-stromal-progenitor cell (BCSP) populations. SSCs are defined as CD45-Ter119-Tie2-CD90-6c3-CD105-CD51+[CD200+]. CD200 was not used in this panel since all SSCs (CD45-Ter119-Tie2-CD90-6c3-CD105-CD51+) in adult mice are CD200+ as shown in our work related to reference (17). **(b)** Flow cytometric based quantification of transient BCSP (CD45-Ter119-Tie2-CD90-6c3-CD105+CD51+) in femurs of experimental groups. n=8 biologically independent mice for Placebo d-28 group, n=7 for GC d-28 group, n=5 for Placebo d-56 group, n=3 for GC d-56 group, n=6 for GC removal and GC+hPTH groups. **(c)** Representative gating strategy for CD31+ endothelial cell populations. **(d)** Representative immunohistochemistry staining for Endomucin (Emcn) in bone marrow of day-28 experimental groups. **(e)** Expression of the glucocorticoid receptor gene *Nr3c1* in SSC-graft derived tissue that underwent single cell RNA-sequencing. **(f)** Matrixplot based on single cell transcriptomic readouts of tissue grafts from mouse SSCs either exposed to placebo, GC, human PTH or GC+human PTH (hPTH 1-34) displaying expression of selected genes for SSPC cluster. All plotted data shown as mean ± SEM. Statistical testing between Placebo and other group by two-sided unpaired student t-test. *p<0.05, **p<0.01, ***p<0.001, ****p<0.0001. Scale bars, 20µm.

Supplementary Figure 4

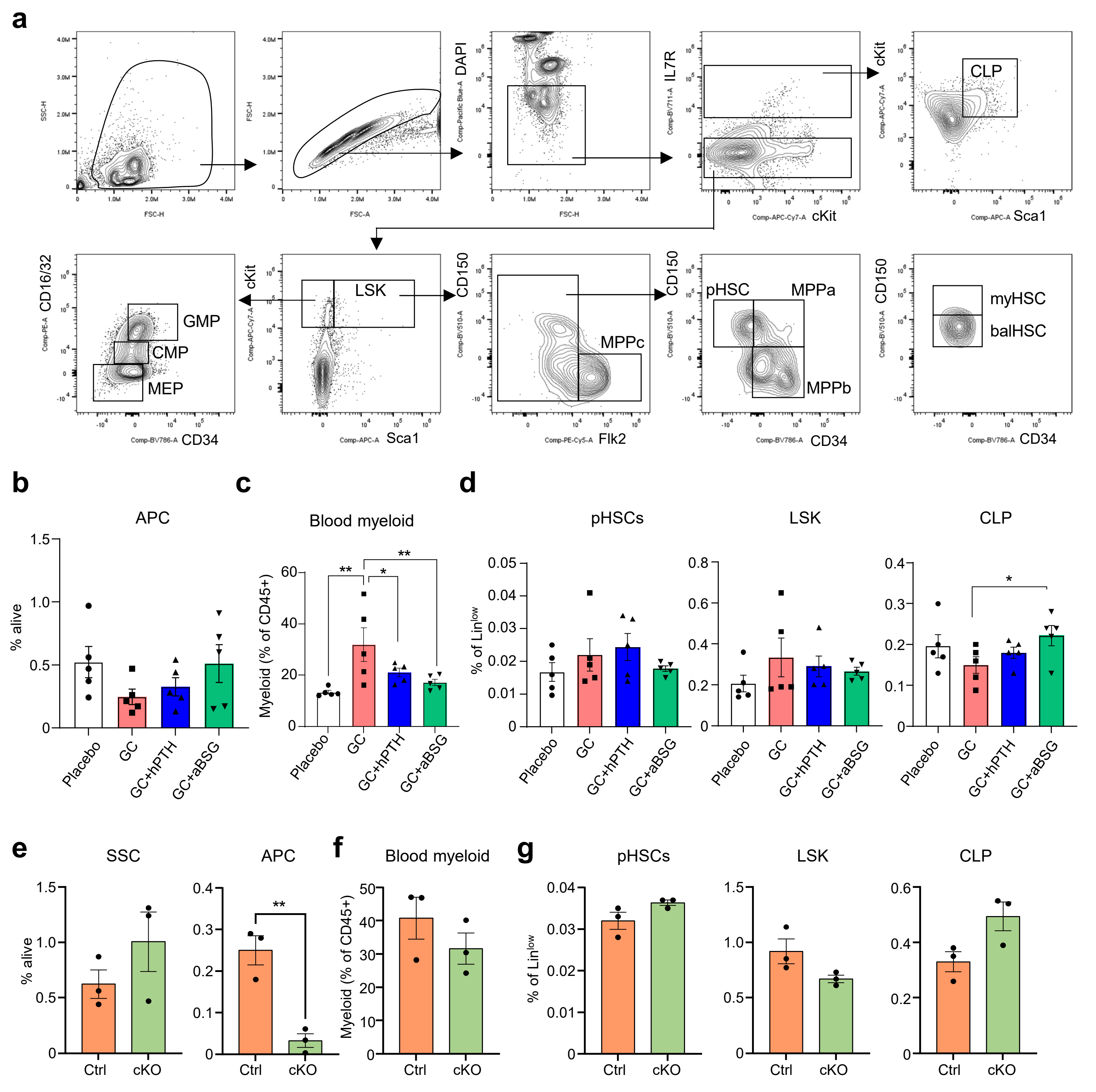

Supplementary Figure 4. Cellular composition of bone marrow and blood in anti-Basigin treated mice. (a) Representative gating strategy for hematopoietic stem and progenitor cells. CLP: common lymphoid progenitor, GMP: granulo-monocyte progenitor, CMP: common myeloid progenitor, MEP: myelo-erythroid progenitor, LSK: Lin^{low}Sca1+cKit+ hematopoietic stem and progenitor cells, MPPa: multipotent progenitor a, MPPb: multipotent progenitor b, MPPc: multipotent progenitor c, pHSC: phenotypic hematopoietic stem cell, myHSC: myeloid skewed HSC, balHSC: balanced HSC. **(b)** Frequency of adipogenic (APC, Lin-Sca1+CD24-) progenitor cells in bone marrow. n=5 biologically independent mice per group. **(c)** Myeloid cell fraction in mouse blood samples. n=5 biologically independent mice per group. **(d)** Flow cytometric analysis of hematopoietic stem and progenitor cells in bone marrow. n=5 biologically independent mice per group. **(e)** Frequency of SSCs and APCs in conditional knockout mice exposed to GC. n=3 biologically independent mice per group. **(f)** Myeloid cell fraction in blood samples of same mice. n=3 biologically independent mice per group. **(g)** Flow cytometric analysis of hematopoietic stem and progenitor cells in bone marrow of same mice. n=3 biologically independent mice per group. All data shown as mean ± SEM. Statistical testing by one-way ANOVA with Fisher-LSD test for pharmacological approach (b-d) or by two-sided unpaired student t-test for comparison of genetic knockout groups (e-g). *p<0.05, **p<0.01, ***p<0.001, ****p<0.0001.

Supplementary Figure 5

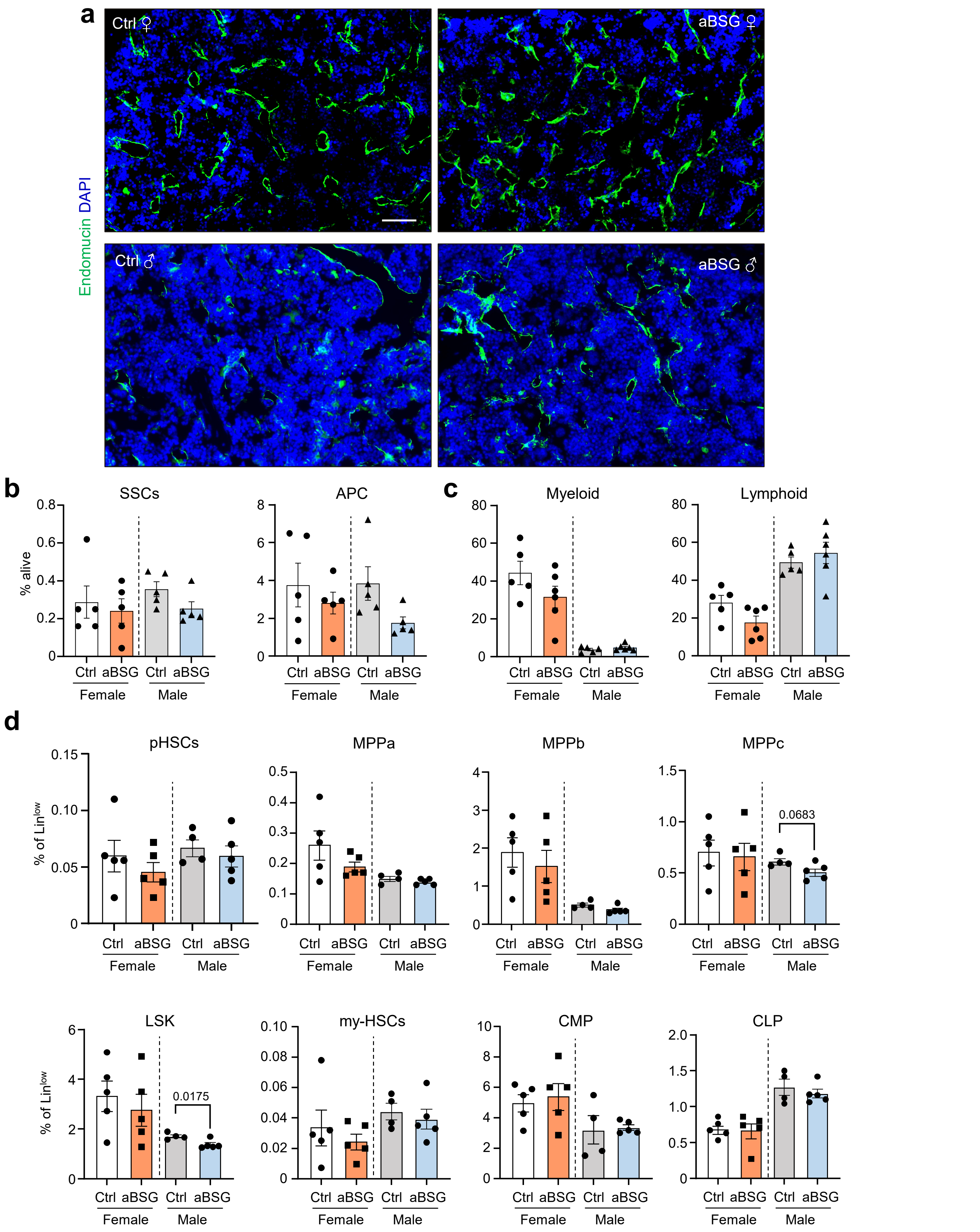

Supplementary Figure 5. Cellular composition of bone marrow in anti-Basigin treated aged mice. (a) Representative immunohistochemistry of endothelial Endomucin expression (green) bone marrow of aged female and male mice treated with control IgG or anti-Basigin. **(b)** Flow cytometric analysis of skeletal cell populations in female and male mice. n=5 biologically independent mice per group. **(c)** Flow cytometric analysis of myeloid and lymphoid cell frequency in blood samples. n=5 biologically independent mice for female and male control groups. n=6 for female and male aBSG groups. **(d)** Flow cytometric analysis of hematopoietic stem and progenitor cell populations in bone marrow. n=5 biologically independent mice for female groups and male aBSG group. n=4 for male control group. All data shown as mean ± SEM. Statistical testing between Placebo and other group by two-sided unpaired student t-test. *p<0.05, **p<0.01, ***p<0.001, ****p<0.0001. Scale bar, 100μm.
