# Supplementary material for: Basigin links altered skeletal stem cell lineage dynamics with glucocorticoid-induced bone loss and impaired angiogenesis
Source: Nat Commun. 2025 Aug 15;16:7606. doi: 10.1038/s41467-025-62881-w (PMC12356905; doi:10.1038/s41467-025-62881-w)
Supplement: Supplementary file 2 — Reporting Summary [file 41467_2025_62881_MOESM2_ESM.pdf]

nature portfolio

Corresponding author(s): Thomas Ambrosi, Nancy Lane

Last updated by author(s): Jul 25, 2025

# Reporting Summary

Nature Portfolio wishes to improve the reproducibility of the work that we publish. This form provides structure for consistency and transparency in reporting. For further information on Nature Portfolio policies, see our Editorial Policies and the Editorial Policy Checklist.

## Statistics

For all statistical analyses, confirm that the following items are present in the figure legend, table legend, main text, or Methods section.

| n/a | Confirmed | |
|---|---|---|
| ☐ | ☒ | The exact sample size (*n*) for each experimental group/condition, given as a discrete number and unit of measurement |
| ☐ | ☒ | A statement on whether measurements were taken from distinct samples or whether the same sample was measured repeatedly |
| ☐ | ☒ | The statistical test(s) used AND whether they are one- or two-sided<br>*Only common tests should be described solely by name; describe more complex techniques in the Methods section.* |
| ☒ | ☐ | A description of all covariates tested |
| ☐ | ☒ | A description of any assumptions or corrections, such as tests of normality and adjustment for multiple comparisons |
| ☐ | ☒ | A full description of the statistical parameters including central tendency (e.g. means) or other basic estimates (e.g. regression coefficient) AND variation (e.g. standard deviation) or associated estimates of uncertainty (e.g. confidence intervals) |
| ☒ | ☐ | For null hypothesis testing, the test statistic (e.g. *F*, *t*, *r*) with confidence intervals, effect sizes, degrees of freedom and *P* value noted<br>*Give P values as exact values whenever suitable.* |
| ☒ | ☐ | For Bayesian analysis, information on the choice of priors and Markov chain Monte Carlo settings |
| ☒ | ☐ | For hierarchical and complex designs, identification of the appropriate level for tests and full reporting of outcomes |
| ☒ | ☐ | Estimates of effect sizes (e.g. Cohen's *d*, Pearson's *r*), indicating how they were calculated |

*Our web collection on statistics for biologists contains articles on many of the points above.*

## Software and code

Policy information about availability of computer code

| | |
|---|---|
| Data collection | BD Diva software and CYTEK SpectroFlow were used for flow cytometry data acquisition. Spectrometric measurements were conducted on a Ultraspec 2100 UV/Visible Spectrophotometer (Biochrom, Harvard Bioscience). Micro-CT Bruker Skyscan 1276 (Bruker Preclinical Imaging) and X-ray scanner (Mozart®, Kubtec Medical Imaging) were used to collect data. |
| Data analysis | Statistical analysis was performed with Prism 10 (GraphPad); ImageJ v1.48 (NIH, http://imagej.nih.gov/ij/); MicroCT analysis with CTAn v1.17.7.2 and CTvox software v3.3.0 (Bruker); FACS analysis was conducted using FlowJo v10 software; 10X Genomics single cell RNA-sequencing data were UMI-collapsed with the Cellranger toolkit version 7.1.0 (10X Genomics Inc). We used the Scanpy package (v.1.9.1.) to explore the data. |

For manuscripts utilizing custom algorithms or software that are central to the research but not yet described in published literature, software must be made available to editors and reviewers. We strongly encourage code deposition in a community repository (e.g. GitHub). See the Nature Portfolio guidelines for submitting code & software for further information.

## Data

Policy information about availability of data

All manuscripts must include a data availability statement. This statement should provide the following information, where applicable:

- Accession codes, unique identifiers, or web links for publicly available datasets
- A description of any restrictions on data availability
- For clinical datasets or third party data, please ensure that the statement adheres to our policy

Source data are provided with this paper. All sequencing data have been submitted to repositories and are available online. Single cell RNA-sequencing data is available from the NCBI Gene expression Omnibus with GEO Accessions GSE253044.

## Research involving human participants, their data, or biological material

Policy information about studies with human participants or human data. See also policy information about sex, gender (identity/presentation), and sexual orientation and race, ethnicity and racism.

| | |
|---|---|
| Reporting on sex and gender | No restrictions were made regarding the sex or gender of the specimen's donor. Patient donor tissue was de-identified and all data was collected aggregated from donors without distinction of sex. |
| Reporting on race, ethnicity, or other socially relevant groupings | No restrictions were made regarding the race and ethnicity of the specimen's donor. |
| Population characteristics | No restrictions were made regarding the age of the specimen's donor. |
| Recruitment | Informed consent was not required as samples from de-identified donors were considered biological waste. Samples were collected and used as they became available. |
| Ethics oversight | Procurement and handling were in accordance with the guidelines set by the Stanford University Institutional Review Board (IRB-35711) and the UC Davis Institutional Review Board (IRB-1997852). |

Note that full information on the approval of the study protocol must also be provided in the manuscript.

# Field-specific reporting

Please select the one below that is the best fit for your research. If you are not sure, read the appropriate sections before making your selection.

[x] Life sciences [ ] Behavioural & social sciences [ ] Ecological, evolutionary & environmental sciences

For a reference copy of the document with all sections, see nature.com/documents/nr-reporting-summary-flat.pdf

# Life sciences study design

All studies must disclose on these points even when the disclosure is negative.

| | |
|---|---|
| Sample size | No statistical test was used to pre-determine the sample sizes. Sample sizes were based on experience from previous work with assays performed. For assays with commonly high variability we typically used n≥5 and for assays with commonly low variability we typically used n<5. |
| Data exclusions | No data of experimental groups were excluded |
| Replication | All data presented are biological replicates unless otherwise stated in the figure legends. Each experimental finding was reproduced in at least two independent biological replicates. |
| Randomization | Animals were allocated randomly into the different experimental group. |
| Blinding | Investigators were partially blinded during data collection and analysis. Controls and samples were treated equally. |

# Reporting for specific materials, systems and methods

We require information from authors about some types of materials, experimental systems and methods used in many studies. Here, indicate whether each material, system or method listed is relevant to your study. If you are not sure if a list item applies to your research, read the appropriate section before selecting a response.

## Materials & experimental systems

| n/a | Involved in the study |
| --- | --- |
| ☐ | ☒ Antibodies |
| ☐ | ☒ Eukaryotic cell lines |
| ☒ | ☐ Palaeontology and archaeology |
| ☐ | ☒ Animals and other organisms |
| ☒ | ☐ Clinical data |
| ☒ | ☐ Dual use research of concern |
| ☒ | ☐ Plants |

## Methods

| n/a | Involved in the study |
| --- | --- |
| ☒ | ☐ ChIP-seq |
| ☐ | ☒ Flow cytometry |
| ☒ | ☐ MRI-based neuroimaging |

# Antibodies

Antibodies used

Flow cytometry: CD90.1 (Thermo Fisher, 47–0900), CD90.2 (Thermo Fisher, 47–0902), CD105 (Thermo Fisher, 13–1051), CD51 (BD Biosciences, 551187), CD45 (BioLegend, 103110), Ter119 (Thermo Fisher, 15–5921), Tie2 (Thermo Fisher, 14–5987), 6C3 (BioLegend, 108312), streptavidin PE-Cy7 (Thermo Fisher, 25–4317), Sca-1 (Thermo Fisher, 56-5981), CD45 (Thermo Fisher, 11–0451), CD31 (Thermo Fisher, 12-0311) and CD24 (Thermo Fisher, 47–0242). CD45 (BioLegend, 304029), CD235a (BioLegend, 306612), CD31 (Thermo Fisher Scientific, 13-0319), CD202b (TIE-2) (BioLegend, 334204), streptavidin APC-AlexaFlour750 (Thermo Fisher, SA1027), CD146 (BioLegend, 342010), PDPN (Thermo Fisher Scientific, 17-9381), CD164 (BioLegend, 324808) and CD73 (BioLegend, 344016).Ter119-PE-Cy5 (116210, BioLegend), CD45-FITC (11-0451, Invitrogen), B220-APC-Cy7 (103224, BioLegend), CD11b-PE-Cy7 (101216, BioLegend), CD3-APC (100236, BioLegend), Gr1-BV711 (108443, BioLegend). Lineage cocktail-Pacific Blue (133310, BioLegend), CD127-BV711 (135035, BioLegend), CD117-APC-Cy7 (105826, BioLegend), Sca1-APC (160904, BioLegend), CD16/32-PE (156606, BioLegend), CD34-BV786 (742971, BD Biosciences), CD135-PE-Cy5 (135312, BioLegend), CD150-BV510 (115929, BioLegend).

In vivo pharmacological studies: aBSG or IgG controls (ThermoFisher, cat#:16-1471-82 & 16-4321-82

IHC: Endomucin, cat# sc-65495, Santa Cruz; CD31: cat# AF3628, Thermofisher; Basigin, cat# 16-1471-82, Thermofisher & cat# NB500-430, Novus Biologicals. Secondary antibodies: AF488 dk anti-rat: Donkey anti-Rat IgG (H+L) Highly Cross-Adsorbed Secondary Antibody, Alexa Fluor™ 488, cat# A21208; Donkey anti-Goat IgG (H+L) Cross-Adsorbed Secondary Antibody, Alexa Fluor™ 647, cat# A21447, both Thermofisher

Validation

All antibodies used are validated, commercially available products. Flow cytometry antibodies have also been validated in previously published studies (e.g. PMID: 29748647 & PMID: 15967997).

# Eukaryotic cell lines

Policy information about cell lines and Sex and Gender in Research

Cell line source(s)

VeraVec HUVEC endothelial cell line (from female patient) provided by collaborator/co-author Fernando Fierro's lab.

Authentication

Commercially available and established cell line, no additional authentication conducted.

Mycoplasma contamination

Cell line was confirmed to be negative for mycoplasma contamination

Commonly misidentified lines
(See ICLAC register)

none

# Animals and other research organisms

Policy information about studies involving animals; ARRIVE guidelines recommended for reporting animal research, and Sex and Gender in Research

Laboratory animals

To study the effects of glucocorticoids on bone biology of young adult mice, experiments were conducted using 3-month-old Balb/cJ male mice, purchased from Jackson Laboratories (Strain#:000651). Pharmacological rescue experiments were conducted with aged (24-months) male and female C57BL/6 mice ordered from NIA . For renal capsule transplantation experiments cells from three-month old male GFP reporter mice (C57BL/6-Tg(CAG-EGFP)1Osb/J; JAX: 003291) were transplanted into male B6 mice (C57BL/Ka-Thy1.1-CD45.1; JAX: 000406). Three-month old immunodeficient NSG mice (NOD.Cg-Prkdcscid Il2rgtm1Wjl/SzJ; JAX: 005557) were used for human cell transplantation studies. Bsgflox/flox mice were obtained from the lab of Dr. Romana Nowak at the University of Illinois at Urbana-Champaign and as previously published. Tamoxifen inducible Col2a1-CreERT2 mice were crossed with Bsgflox/flox mice to generate Col2a1-CreERT2 x Bsgflox/+ (conditional heterozygous Basigin knockout, cKO) female offsprings.

Wild animals

This study did not involve wild animals.

Reporting on sex

Male and female mice were used as indicated and reported disaggregated.

Field-collected samples

This study did not involve samples collected from the field.

| | |
|---|---|
| Ethics oversight | All mouse experiments complied with all relevant ethical regulations and were conducted under approved protocols by the Institutional Animal Care and Use Committee of Stanford University and the University of California Davis. Mice were maintained at animal facilities in accordance with institutional guidelines and procedures were conducted under valid IACUC protocols. |

Note that full information on the approval of the study protocol must also be provided in the manuscript.

# Plants

| | |
|---|---|
| Seed stocks | No plants were used for this study |
| Novel plant genotypes | No plants were used for this study |
| Authentication | No plants were used for this study |

# Flow Cytometry

## Plots

Confirm that:

- [x] The axis labels state the marker and fluorochrome used (e.g. CD4-FITC).
- [x] The axis scales are clearly visible. Include numbers along axes only for bottom left plot of group (a 'group' is an analysis of identical markers).
- [x] All plots are contour plots with outliers or pseudocolor plots.
- [x] A numerical value for number of cells or percentage (with statistics) is provided.

## Methodology

| | |
|---|---|
| Sample preparation | Femur bones were dissected, cleaned of soft tissue and crushed using mortar and pestle. Then, the tissue was digested in M199 (cat#11150067, Thermo Fisher Scientific) with 2.2 mg/ml collagenase II buffer (cat#C6885, Sigma-Aldrich) at 37 °C for 60 min. Dissociated cells were strained through a 100-µm nylon filter, washed in staining medium (10% fetal bovine serum (FBS) in PBS) and pelleted at 200g at 4°C. The cell pellet was resuspended in staining medium and red blood cells were depleted via ACK lysis for 5 min. The cells were washed again in staining medium and pelleted at 200g at 4°C. Then, the cells were prepared for flow cytometry with fluorochrome-conjugated antibodies. Blood samples were ACK lysed and stained with antibodies as described in the methods. |
| Instrument | FACS Aria II Instrument (BD BioSciences) and CYTEK Aurora CS (CYTEK) |
| Software | Flow cytometric analysis was conducted using FlowJo (FLOWJ LLC, v10.10) |
| Cell population abundance | Cell abundance was reported as percentage of cell population of interest based on percentage of alive cells (mesenchymal cells), percentage of CD45+ fraction (blood) and percentage of Lineage-low (bone marrow). Cells for functional experiments were sorted in purity mode. All gating was based on previous literature and published work by our group. Cell population abundance was in the same range as expected from previous work. |
| Gating strategy | All gating strategies are provided with Supplementary information. Forward/Sideward scatter was used to gate out debris and multiplets. DAPI was used as live/dead stain. All gates were based on fluorescence-minus-one (FMO) controls to separate positive from negative fractions. |

- [x] Tick this box to confirm that a figure exemplifying the gating strategy is provided in the Supplementary Information.
